# Structural Changes Induced by Quinones: High‐Resolution Microwave Study of 1,4‐Naphthoquinone

**DOI:** 10.1002/cphc.202000665

**Published:** 2020-11-13

**Authors:** Shefali Saxena, Sanjana Panchagnula, M. Eugenia Sanz, Cristóbal Pérez, Luca Evangelisti, Brooks H. Pate

**Affiliations:** ^1^ Department of Chemistry King's College London London United Kingdom; ^2^ Department of Chemistry University of Virginia Charlottesville VA USA; ^3^ Department of Chemistry “G. Ciamician” University of Bologna Via Selmi 2 Bologna 40126 Italy

**Keywords:** PAH, quantum-chemistry calculations, quinones, rotational spectroscopy, structural determination

## Abstract

1,4‐Naphthoquinone (1,4‐NQ) is an important product of naphthalene oxidation, and it appears as a motif in many biologically active compounds. We have investigated the structure of 1,4‐NQ using chirped‐pulse Fourier transform microwave spectroscopy and quantum chemistry calculations. The rotational spectra of the parent species, and its ^13^C and ^18^O isotopologues were observed in natural abundance, and their spectroscopic parameters were obtained. This allowed the determination of the substitution *r*_s_, mass‐weighted *r*_m_ and semi‐experimental *r*_e_^SE^ structures of 1,4‐NQ. The obtained structural parameters show that the quinone moiety mainly changes the structure of the benzene ring where it is inserted, modifying the C−C bonds to having predominantly single or double bond character. Furthermore, the molecular electrostatic surface potential reveals that the quinone ring becomes electron deficient while the benzene ring remains a nucleophile. The most electrophilic areas are the hydrogens attached to the double bond in the quinone ring. Knowledge of the nucleophilic and electrophilic areas in 1,4‐NQ will help understanding its behaviour interacting with other molecules and guide modifications to tune its properties.

## Graphical abstract

**The structure of 1,4‐naphthoquinone** has been investigated by rotational spectroscopy in combination with quantum‐chemistry calculations. The determined structural parameters show that 1,4‐naphthoquinone behaves as a combination of benzene and para‐benzoquinone units. The quinone moiety changes the structure of the benzene ring in which it is inserted and makes it electron deficient.

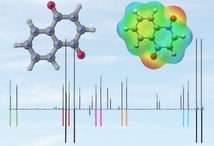

## 1 Introduction

Naphthoquinones are derivatives of naphthalene containing two carbonyl groups, and as such, a class of oxygenated polycyclic aromatic hydrocarbons (PAHs). They are naturally present in a variety of plants,^[1]^ and of great interest in chemistry and biology. Their biological activity is linked to their ability to participate in redox processes by accepting one or two electrons thereby forming radicals and anionic species (semiquinones and hydroquinones) that can participate in further reactions.^[2]^ Biochemically, they also act as electrophiles, reacting with nucleophiles in the biological medium and forming covalent bonds. Naphthoquinones have been found to have antibacterial,^[3]^ antifungal,^[4]^ and anti‐tumour_,_[^5^, ^6^] effects, which has led to their use as drugs and as scaffolds to develop new medicines.^[7]^ In the atmosphere, naphthoquinones appear as the result of incomplete combustion of fuels^[8]^ and in cigarette smoke.^[9]^ They can also form through reaction of naphthalene with OH and NO_3_ radicals,[^10^, ^11^, ^12^] and by photolysis of other species.^[13]^ They have been identified as major contributors to air pollution.[^2^, ^14^] Their toxicity is higher than that of naphthalene,^[15]^ which is one of the most abundant PAHs in the atmosphere.[^16^, ^17^]

**Figure 1:**
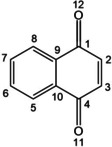
Scheme of the molecular structure of 1,4‐naphthoquinone and labelling of carbon and oxygen atoms.

1,4‐naphthoquinone (1,4‐NQ, see Figure 1) is the major structural component of vitamin K,^[18]^ and has been found to strongly inhibit cancer cell growth.^[19]^ It is a major scaffold to develop new compounds with tuned features, by modifying its structural and electronic properties. In spite of this, there are very limited spectroscopic investigations focusing on this species. The effect of 1,4‐NQ on cell membrane models was studied by vibrational spectroscopy.^[20]^ The interactions of 1,4‐NQ with DNA have been investigated by Raman and Surface Enhanced Raman spectroscopy^[21]^ to determine possible binding sites. However, in none of these studies detailed information at the atomic level was obtained. Understanding the specificity of 1,4‐NQ against biomolecular targets and its reaction mechanisms in the atmosphere requires precise spectroscopic characterisation of its molecular structure and electronic properties.

Here we present the structural study of 1,4‐NQ using chirped‐pulse Fourier transform microwave spectroscopy (CP‐FTMW).^[22]^ Rotational spectroscopy is a high‐resolution spectroscopic technique sensitive to minute structural changes and therefore ideally suited to structural studies, with applications in biomolecular characterisation, astrochemistry and atmospheric chemistry.[^23^, ^24^, ^25^, ^26^, ^27^, ^28^, ^29^, ^30^] Nowadays rotational spectroscopy is combined with supersonic jets, which provide a collisionless environment where molecules are virtually isolated and free from any of the interactions with the solvent or lattice constraints that occur in condensed phases. Rotational spectroscopy data can then be compared directly with that from *in vacuo* theoretical calculations and used to benchmark the accuracy of different theoretical methods.

The rotational spectra of the parent and heavy atom isotopologues of 1,4‐NQ have been observed in natural abundance and analysed. The substitution, mass‐weighted and semi‐experimental structures of 1,4‐NQ have been determined and compared with those predicted by theoretical methods as well as with the experimental X‐ray structure. The 1,4‐NQ structural parameters show that it can be described as a combination of benzene and para‐benzoquinone moieties. Comparison with naphthalene reveals that the introduction of a quinone moiety in 1,4‐NQ results in significant changes in structural parameters and electronic density.

## 2 Results and Discussion

### 2.1 Rotational Spectrum

To aid spectral assignments, we performed calculations to optimise the geometry of 1,4‐NQ, and predict its rotational constants and dipole moment components using Gaussian.^[31]^ The theoretical parameters from ab initio second order Møller‐Plesset perturbation theory, and the density functionals B3LYP as well as M06‐2X, with the 6‐311++G(d,p) basis set, are presented in Table 1.

**Table 1:** Theoretical spectroscopic parameters of 1,4‐NQ using the 6–311++G(d,p) basis set.

Parameters	B3LYP	MP2	M062X
*A* ^[a]^ (MHz)	1326.1	1315.4	1336.4
*B* (MHz)	1092.4	1089.5	1095.8
*C* (MHz)	599.0	595.9	602.1
*μ*_a_/*μ*_b_/*μ*_c_^[b]^ (D)	1.5/0.0/0.0	1.3/0.0/0.0	1.4/0.0/0.0

[a] *A*, *B* and *C* are the rotational constants. [b] *μ*_a_, *μ*_b_ and *μ*_c_ are the electric dipole moment components along the principal inertial axes *a*, *b* and *c*.

1,4‐NQ is an oblate asymmetric top with its dipole moment along the *a* principal inertial axis. Hence, we searched for patterns involving the usually strong R‐branch *a*‐type transitions with *K*_‐1_=0, 1 in the 2–8 GHz broadband rotational spectrum. Transitions from the parent isotopologue were very intense and easily located. The measured transitions were fitted using Watson's S‐reduced Hamiltonian in the III^*l*^ representation and Pickett's programs.^[32]^ The experimental rotational and centrifugal distortion constants are shown in the second column of Table 2.

**Table 2:** Experimental spectroscopic parameters of the parent, ^13^C and ^18^O isotopologues of 1,4‐NQ.

Parameter	Parent	^13^C_1/4_	^13^C_2/3_	^13^C_7/6_	^13^C_8/5_	^13^C_9/10_	^18^O
*A* ^[a]^ (MHz)	1328.29178(21)^[b]^	1321.06913(24)	1326.82916(27)	1326.73795(23)	1321.68409(38)	1326.62360(25)	1281.97853(52)
*B* (MHz)	1092.80549(16)	1090.420385(55)	1080.863012(59)	1075.855178(49)	1087.468822(85)	1092.652974(57)	1086.82929(13)
*C* (MHz)	599.85917(14)	597.658463(35)	595.942374(38)	594.396842(32)	596.897085(55)	599.465989(36)	588.476578(79)
*D*_J_ (kHz)	0.0324(34)	[0.0324]^[f]^	[0.0324]	[0.0324]	[0.0324]	[0.0324]	[0.0324]
*D*_JK_ (kHz)	−0.0482(43)	[−0.0482]	[−0.0482]	[−0.0482]	[−0.0482]	[−0.0482]	[−0.0482]
*D*_K_ (kHz)	0.0156(31)	[0.0156]	[0.0156]	[0.0156]	[0.0156]	[0.0156]	[0.0156]
*P*_c_ (uÅ^2^)	0.21342(10)	0.21321(5)	0.21442(5)	0.21321(4)	0.21387(7)	0.20910(5)	0.21453(10)
a/b/c^[c]^ (D)	y/n/n	y/n/n	y/n/n	y/n/n	y/n/n	y/n/n	y/n/n
N^[d]^	44	21	21	21	21	21	21
σ^[e]^ (kHz)	1.9	0.7	0.7	0.6	1.0	0.7	1.5

^[a]^ *A*, *B* and *C* are the rotational constants. ^[b]^ Standard error in parentheses in the units of the last digit. ^[c]^ Yes (y) or no (n) observation of *μ*_a_‐, *μ*_b_‐ and *μ*_c_‐type transitions. ^[d]^ Number of rotational transitions included in the fit. ^[e]^ Rms deviation of the fit. ^[f]^ Values fixed to those of the parent species.

**Figure 2:**
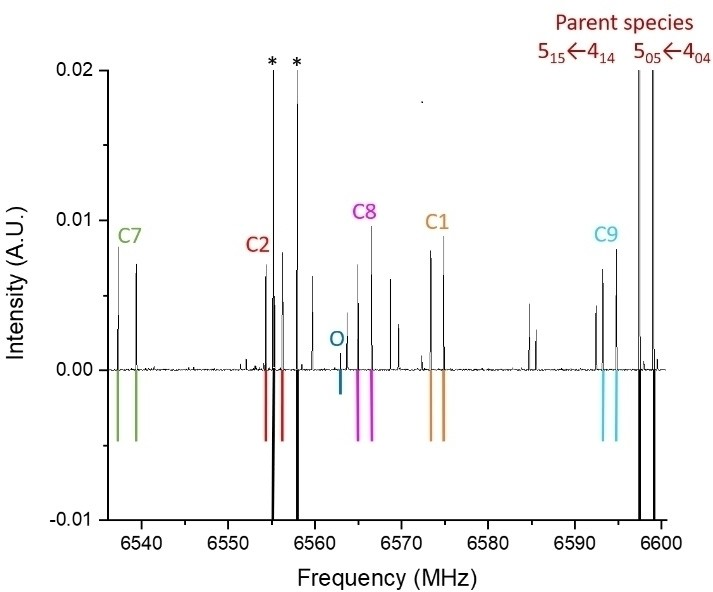
A section of the experimental (top, in black) and simulated (bottom, in colour) rotational spectrum of 1,4‐NQ showing transitions from the parent species, and the ^13^C and ^18^O isotopologues in natural abundance. The 5_1,5_←4_1,4_ and 5_0,5_←4_0,4_ transitions are shown for parent and ^13^C species, the 4_1,3_←3_1,2_ transition is shown for the ^18^O species. The asterisks indicate other transitions of the parent species.

Transitions corresponding to ^13^C and ^18^O isotopologues were observed at the expected shifts below the frequencies from the parent species (see example in Figure 2). Due to the presence of a C_2_ symmetry axis in 1,4‐NQ, there are only five distinct ^13^C and one ^18^O isotopologues. Each ^13^C and ^18^O isotopologue transition exhibited an intensity about twice the natural abundance of the corresponding isotope. Isotopologue transitions were fit to the same Hamiltonian as the parent species, which yielded the experimental rotational constants of Table 2.

### 2.2 Inertial Defect

Upon isotopic substitution of carbons and oxygen in 1,4‐NQ, the planar moment *P*_c_ are nearly invariant (see Table 2), which shows that all the heavy atoms are located on the *ab* inertial plane and therefore 1,4‐NQ is planar.

Generally, the inertial defect for a planar rigid molecule is expected to be close to zero and have a small, positive value. However, it has been observed that PAHs usually have a small negative value of the inertial defect due to the dominant effect of low‐frequency out‐of‐plane vibrations.[^33^, ^34^, ^35^] The value of 1,4‐NQ inertial defect is Δ=*I*_c_−*I*_a_−*I*_b_=−0.4268(2) uÅ^2^. The value obtained for 1,4‐NQ can be compared with some of similar systems such as naphthalene (−0.137 uÅ^2^),^[35]^ azulene (−0.152 uÅ^2^),^[36]^ trans‐1‐hydroxynaphthalene (−0.212 uÅ^2^),^[37]^ cis‐2‐hydroxynaphthalene (−0.237 uÅ^2^),^[37]^ cis‐1‐naphthaldehyde (−0.413 uÅ^2^),^[37]^ trans‐2‐naphthaldehyde (−0.366 uÅ^2^),^[37]^ coumarin (−0.294 uÅ^2^)^[38]^ and 9‐fluorenone (−0.36670(32) uÅ^2^).^[39]^ On comparison, aromatic molecules possessing carbonyl functional groups seem to exhibit more negative inertial defects.

The small negative inertial defects of PAHs have been rationalised in terms of their out‐plane vibrations. Oka proposed an empirical expression to calculate the inertial defect of planar molecules based on their lowest out‐of‐plane vibration, predicted theoretically.[^33^, ^34^, ^35^] The expression has been recently refined for polycyclic aromatic molecules. Gruet *et al*.^[40]^ proposed to use a different value of α in Oka's empirical formula, maintaining the dependence on the lowest out‐of‐plane vibration. Jahn *et al*.^[35]^ proposed another value of α, and to include as many low‐ frequency out‐of‐plane vibrations as the number of rings in the PAH, or the number of rings plus one^[35]^ for those PAHs with very low out‐of‐plane vibrations.

To our knowledge, there is no experimental data on the low frequency modes of 1,4‐NQ. Anharmonic frequency calculations of PAHs can be modelled at a relatively low computational cost and considerable accuracy by using density functional theory methods.[^37^, ^40^] Using the B3LYP/6‐311++G(d,p) level of theory, which was found to perform well in related molecules,^[35]^ we have predicted the low‐frequency modes of 1,4‐NQ to be at 78 cm^−1^ and 119 cm^−1^ (harmonic) and 79 cm^−1^ and 120 cm^−1^ (anharmonic). These values are similar to those measured experimentally for 1‐cis‐naphthaldehyde and 2‐trans‐naphthaldehyde,^[37]^ specially the lowest‐frequency mode. Using the harmonic values, we calculated inertial defects of −0.344 uÅ^2^ and −0.407 uÅ^2^ using the expressions of Gruet *et al*. and Jahn *et al*., respectively. Including the second lower‐frequency mode improves the prediction of 1,4‐NQ inertial defect, bringing it within 5 % of the experimental value, which is comparable to predictions of related PAHs using harmonic frequencies calculated with the same computational method.^[35]^

### 2.3 Structural Determination

The values of the rotational constants of the parent species and observed isotopologues of 1,4‐NQ were used to determine its heavy atom substitution structure (*r*_s_) through Kraitchman's equations,^[41]^ and using the KRA and EVAL programs.^[42]^ From the difference of the moments of inertia of the isotopologues with respect to the parent species, the positions of the corresponding substituted atoms in the principal inertial axis system were obtained. 1,4‐NQ is a planar molecule, and therefore any two pairs of moments of inertia can be used to determine its structure. This can result in slightly different structural parameters, since the difference between the moments of inertia is not exactly zero. In fact, 1,4‐NQ has a relatively large inertial defect of −0.4268(2) uÅ^2^. Therefore, we calculated *r*_s_ atomic coordinates using each pair of rotational constants. The values obtained were all the same within Costain's error (see Table S8), and so were the determined bond lengths and angles. The average values of the structural parameters obtained with each pair of coordinates are shown in Table 3.

**Table 3:** Comparison of the bond lengths (in Å) and angles (in degrees) of the heavy atoms of 1,4‐NQ determined experimentally and theoretically, with those of related molecules.

	1,4‐NQ					2‐methyl‐1,4‐NQ			Naphthalene			1,4‐benzoquinone	
	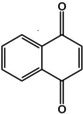					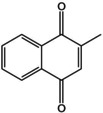			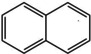			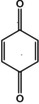	
	*r*_s_	*r*_e_^SE^	*r*_m_	B3LYP	X‐ray^[a]^		Elec. Diff.^[b]^		Elec. Diff.^[c]^	X‐ray^[d]^		Elec. Diff.^[e]^	X‐ray^[f]^
r (C1−C2)	1.488(2)	1.485(2)	1.490(3)	1.484	1.48		1.492(6)		1.381(2)	1.377(2)		1.481(2)	1.477(8)
r (C1−C9)	1.461(5)	1.460(5)	1.481(3)	1.492	1.43		1.485(6)		1.422(3)	1.424(2)		1.481(2)	1.477(8)
r (C8−C9)	1.426(5)	1.411(5)	1.408(4)	1.397	1.39		1.391(2)		1.422(3)	1.424(2)		–	–
r (C7−C8)	1.395(2)	1.397(2)	1.387(3)	1.391	1.41		1.384(2)		1.381(2)	1.377(2)		–	–
r (C2−C3)	1.336(3)	1.320(3)	1.304(7)	1.340	1.31		1.342^[a]^		1.417(4)	1.411(2)		1.344(3)	1.322(8)
r (C6−C7)	1.396(3)	1.412(3)	1.387(8)	1.397	1.37		1.393(3)		1.417(4)	1.411(2)		–	–
r (C9−C10)	1.386(3)	1.414(3)	1.381(8)	1.407	1.39		1.400(3)		1.412(8)	1.421(2)		1.344(3)	1.322(8)
r (C1−O)	1.223(1)	1.220(2)	1.214(3)	1.220	1.21		1.215(4)		–	–		1.225(2)	1.222(8)
∠(C2−C1−C9)	116.8(2)	117.2(2)	–	117.2	121.5		–		–	120.2(1)		118.1(3)	117.48
∠(C1−C9−C10)	121.4(5)	120.6(4)	–	120.5	118.0		–		119.5(3)	119.0(1)		–	–
∠(C8−C9−C10)	119.7(5)	119.9(4)	–	119.8	119.0		–		–	–		–	–
∠(C9−C8−C7)	120.0(2)	119.8(2)	–	120.0	121.0		–		–	–		–	–
∠(C8−C7−C6)	120.2(1)	120.3(1)	–	120.2	119.0		–		–	–		–	–
∠(C1−C2−C3)	121.9(1)	122.2(1)	–	122.2	120.5		–		–	120.5(4)		–	121.06
∠(C2−C1−O)	120.0(2)	120.6(2)	–	120.4	118.5		–		–	–		–	121.06
∠(C9−C1−O)	123.2(2)	122.2(2)	–	122.3	119.0		–		–	–		–	–

[a] Ref. [48] [b] Ref. [49] [c] Ref. [50] [d] Ref. [51] [e] Ref. [52] [f] Ref. [53]

**Figure 3:**
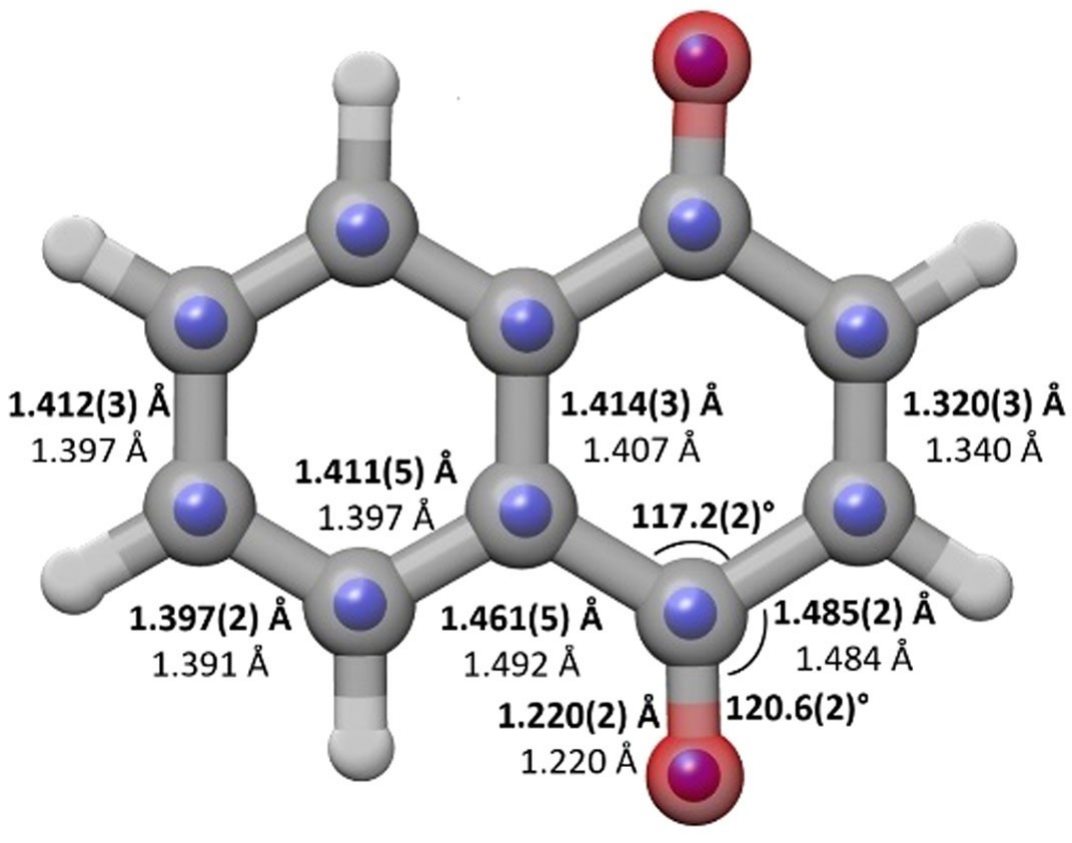
Comparison of the *r_e_* ^*SE*^ structure of 1,4‐NQ (blue spheres) with the B3LYP structure (grey framework). The *r_e_* ^*SE*^ (top) and B3LYP (bottom) values of selected bond lengths and angles of 1,4‐NQ are also indicated on the figure.

The accuracy of the *r*_s_ structure is known to suffer when atoms are close to the principal inertial axes and from distinct vibrational corrections from different isotopologues, particularly in large molecules such as 1,4‐NQ.^[43]^ To address vibrational effects we also determined the semi‐experimental *r*_e_^SE^ structure^,^.[^44^, ^45^] Using the vibrational corrections *B*_e_^calc^−*B*_0_^calc^ from anharmonic calculations of the force field at B3LYP/6‐311++(d,p) level, we corrected the experimental ground state *B*_0_ rotational constants. The resulting semi‐experimental equilibrium constants *B*_e_^SE^ are collected in Table S9. With these constants, the inertial defect of 1,4‐NQ is reduced to −0.0373(2) uÅ^2^, about an order of magnitude lower than the ground state value of −0.4268(2) uÅ^2^. Comparable reductions have been reported for other molecules in the literature.^[46]^ The correction is not perfect, since the value of the inertial defect should now be zero. Therefore, we calculated the *r*_e_^SE^ structure (shown in Table 3) from the average of the structural parameters yielded by the three possible combinations of moments of inertia (see Table S10), following the same procedure as for the *r*_s_ structure. The averaged values of the *r*_e_^SE^ atomic coordinates are shown in an overlay with the theoretical structure in Figure 3.

We also determined the mass‐dependent *r*_m_^(2)^ structure^[47]^ through a least‐squares fit of the experimental ground state moments of inertia of the observed isotopologues, using the program STRFIT.^[42]^ Due to strong correlations between structural parameters, only bond distances could be determined. The bond lengths obtained from the best fit are shown in Table 3, and the fit is collected in Table S11.

The structural parameters obtained from the three structures are compared in Table 3 with the B3LYP *r*_e_ equilibrium structure. If we consider the typical accepted distances of 1.48 Å and 1.34 Å for single and double C−C bonds, respectively, then C2–C3 has a strong double bond character while C1−C2 and C1−C9 have single bond character. The other bond lengths in 1,4‐NQ, except the carbonyl bond, have intermediate character between single and double bonds, with values of 1.40–1.41 Å. The atoms C9 and C10 have the smallest values of the coordinates, and therefore the bond lengths in which they are involved change the most between the different methods. The C−O bond length is typical of carbonyl bond lengths.

The *r*_s_, *r*_m_^(2)^ and *r*_e_^SE^ structures agree on the character of the bonds in 1,4‐NQ. However, the *r*_s_ and *r*_m_^(2)^ structures have some limitations, as highlighted before. The *r*_e_^SE^ structure is generally considered to be the most sophisticated and the one more closely approaching the equilibrium structure, as vibrational contributions from the different isotopologues are accounted for^,^.[^44^, ^45^] Therefore we will consider the *r*_e_^SE^ structure in the discussion that follows. This method has been found to offer reliable results in other structural studies of planar molecules.^[46]^

Our *r*_e_^SE^ bond lengths are in good agreement with the X‐ray data for 1,4‐NQ, except for C1−C9 and C6−C7. However, the X‐ray structure is not symmetric, with the bond lengths differing by up to 0.03 Å. This can be due to packing effects in the crystal or the quality of the X‐ray investigation itself. To our knowledge, there are no other structural studies of 1,4‐NQ. However, a gas phase electron diffraction study of 2‐methyl‐1,4‐naphthoquinone was recently published^[49]^ and the data is also displayed in Table 3. In this study the C2−C3 bond length was fixed to the B3LYP/cc‐pVTZ value, but the other bond lengths show the same character as those determined here, and they are generally in good agreement.

One of the questions in examining the structure of 1,4‐NQ with respect to that of naphthalene is whether the changes caused by having a quinone moiety are localised in the quinone ring or affect both fused rings. Comparison of the structural parameters of 1,4‐NQ with those of naphthalene^,[50,51]^ indicates that the main changes in 1,4‐NQ affect the quinone ring. Specifically, C1−C9 and C1−C2 elongate, and now have single bond character, while C2−C3 shortens and has double bond character. The bond lengths in the quinone part of 1,4‐NQ are quite close to those of para‐benzoquinone (see Table 3), with the exception of the C9−C10 bond, which is much longer in 1,4‐NQ and is intermediate between a single and double bond. In the 1,4‐NQ benzene moiety, the bond lengths show some small variations with respect to naphthalene. Specifically, the C7−C8 bond length increases by +0.016 Å, an amount significantly higher than its experimental uncertainties in 1,4‐NQ and naphthalene. In addition, the *r*_e_^SE^ value for the C8−C9 bond in 1,4‐NQ points to a slight shortening from naphthalene. These changes make the bonds closer to the average C−C bond length of 1.39 Å in benzene.^[54]^ The bond angles in 1,4‐NQ are all close to 120°, with the exceptions of ∠C2−C1−C9 and ∠C1−C2−C3, with more acute and obtuse values, respectively, that match those of p‐benzoquinone. Hence, from our structural data we can conclude that 1,4‐NQ behaves as a combination of benzene and p‐benzoquinone units.

**Figure 4:**
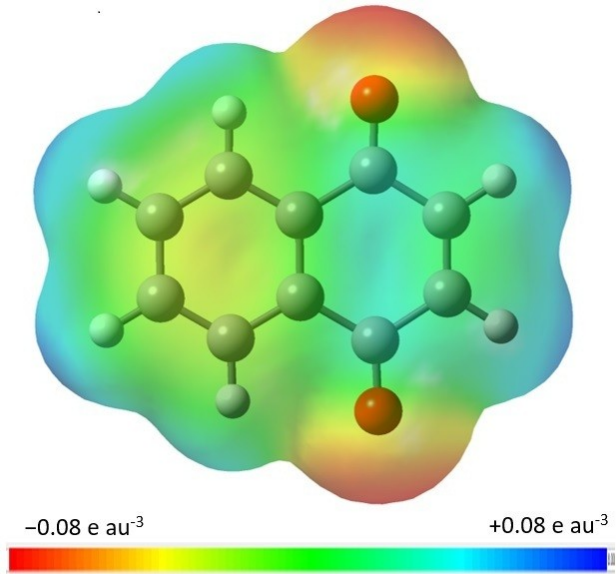
Molecular electrostatic surface potential at the isosurface having an electron density 0.001 e au^−3^.

The changes in the geometric structure of 1,4‐NQ with respect to naphthalene are linked to substantial changes in the electronic structure, as shown in the molecular electrostatic surface potential (see Figure 4). In naphthalene there is considerable electronic density on the benzene rings, making them nucleophiles.^[55]^ The replacement of two −CH groups with two carbonyl groups in 1,4‐NQ decreases the electronic density on the quinone ring, making its centre electrophilic. This is a more dramatic modification than those induced by aldehyde and hydroxyl substituents.^[37]^ In 1‐ and 2‐hydroxynaphthalene the overall electron density on the rings stays mostly the same as in naphthalene, while in 1‐ and 2‐naphthaldehyde it decreases but the rings are still nucleophiles. In 1,4‐NQ, only the benzene ring maintains its nucleophilic character. However, the most nucleophilic areas are, as expected, the carbonyl oxygens, while the most electrophilic areas are the hydrogens bonded to C2 and C3, followed by those bonded to C6 and C7.

The reactivity of 1,4‐NQ can be rationalised by considering the areas of different electronic density revealed by the molecular electrostatic surface potential. For example, it can undergo nucleophilic addition reactions (Michael reactions), where a nucleophile preferentially attacks positions 2 or 3 of 1,4‐NQ^,^.[^56^, ^57^] Cycloadditions and Diels‐Alder reactions of 1,4‐NQ proceed via the same positions. Interactions with electrophiles take place though the carbonyl oxygens. In our study of the interactions of 1,4‐NQ with water,^[58]^ water binds as a hydrogen bond donor to one of the carbonyl oxygens, and prefers to establish secondary interactions with the most electrophilic hydrogens attached to C2 and C3. Research into derivatives of 1,4‐NQ seeks to enhance or reduce the attributes of the different areas to modify preferred reaction sites.

## 3 Conclusions

1,4‐NQ has been investigated using broadband rotational spectroscopy and computational methods. Characterisation of the rotational spectrum of the parent species, and the ^13^C and ^18^O isotopologues allowed determination of the heavy‐atom substitution *r*_s_, semi‐experimental *r*_e_^SE^ and mass‐dependent *r*_m_^(2)^ structures of 1,4‐NQ. The structural parameters of 1,4‐NQ show that it behaves as a combination of benzene and para‐benzoquinone moieties, with the two carbonyl groups introducing structural changes in the benzene ring in which they are inserted. The results presented in this work are the basis to undertake more complex structural studies of 1,4‐NQ interacting with other molecules of atmospheric or astrophysical relevance, such as water or atmospheric radicals, and to explore and explain its behaviour in drug‐related compounds.

## Experimental Section

The rotational spectrum of 1,4‐NQ was recorded using chirped‐pulse Fourier transform microwave spectrometers at King's College London[^59^, ^60^] and University of Virginia.^[61]^ The instruments use an arbitrary waveform generator to produce microwave excitation chirped pulses in the range 2–8 GHz, which are amplified by a travelling wave tube amplifier and broadcast into the vacuum chamber by a broadband microwave horn antenna. 1,4‐NQ (97 %) was purchased from Sigma‐Aldrich and used without further purification. The pure sample is a solid at room temperature with a low vapour pressure, and it was heated to 403.15 K via bespoke heating nozzles to increase its concentration in the gas phase. The vaporised molecules were then seeded in neon as a carrier gas at backing pressures of 1–5 bar and conducted into the vacuum chamber where they form a supersonic jet and interact with the MW excitation field. The molecular emission signals were collected by a broadband MW horn antenna, amplified by a low‐noise amplifier and recorded in the time domain by the digital oscilloscope. A fast Fourier transform algorithm converted the signals from the time domain to the frequency domain. The frequency accuracy of unblended lines is better than 10 kHz. The final spectrum resulted from the averaging of a million FIDs.

## Conflict of interest

The authors declare no conflict of interest.

## Supporting information

As a service to our authors and readers, this journal provides supporting information supplied by the authors. Such materials are peer reviewed and may be re‐organized for online delivery, but are not copy‐edited or typeset. Technical support issues arising from supporting information (other than missing files) should be addressed to the authors.

Supplementary
