# Supplementary material for: Structural Changes Induced by Quinones: High‐Resolution Microwave Study of 1,4‐Naphthoquinone
Source: Chemphyschem. 2020 Nov 13;21(23):2579–84. doi: 10.1002/cphc.202000665 (PMC7756206; doi:10.1002/cphc.202000665)
Supplement: Supplementary file 1 — Supplementary [file CPHC-21-2579-s001.pdf]

ChemPhysChem

Supporting Information

Structural Changes Induced by Quinones: High-Resolution Microwave Study of 1,4-Naphthoquinone

Shefali Saxena, Sanjana Panchagnula, M. Eugenia Sanz,* Cristóbal Pérez, Luca Evangelisti, and Brooks H. Pate

Supporting Information

Table of Contents

Tables S1 – S7 Measured frequencies of parent and isotopic species..**3**

Table S8 Average values of r_s atomic coordinates of 1,4-NQ..**7**

Table S9 Calculation of semi-experimental equilibrium rotational constants B_e^{SE} of 1,4-NQ **7**

Table S10 Average values of r_e^{SE} atomic coordinates of 1,4-NQ **7**

Table S11 Abbreviated STRFIT output for the r_m structural fit of 1,4-NQ **8**

Table S12 Cartesian coordinates of the optimized geometry of 1,4-NQ **12**

Table S1. Measured frequencies and residuals (MHz) of the rotational transitions of parent 1,4-naphthoquinone (1,4-NQ).

J'	K'_{-1}	K'_{+1}	*J"*	K''_{-1}	K''_{+1}	ν_{obs}	ν_{obs}- ν_{calc}
4	2	2	4	2	3	2466.111	0.001
3	1	2	3	1	3	2787.477	0.002
3	3	1	3	1	2	2878.437	-0.001
2	1	2	1	1	1	2892.361	0.000
7	4	3	7	4	4	3003.726	-0.002
2	0	2	1	0	1	3061.544	0.000
3	2	2	3	0	3	3102.662	0.001
4	3	2	4	1	3	3328.290	0.001
6	3	3	6	3	4	3541.535	0.005
2	1	1	1	1	0	3878.267	0.001
5	2	3	5	2	4	3909.311	0.001
4	1	3	4	1	4	4105.245	0.001
3	1	3	2	1	2	4172.865	0.000
4	2	3	4	0	4	4188.537	0.003
5	3	3	5	1	4	4219.198	0.000
3	0	3	2	0	2	4226.930	-0.000
7	3	4	7	3	5	5046.715	-0.002
3	2	2	2	2	1	5077.971	0.001
6	2	4	6	2	5	5248.875	-0.001
6	3	4	6	1	5	5327.905	-0.002
5	1	4	5	1	5	5345.506	0.000
5	2	4	5	0	5	5361.962	0.002
4	1	4	3	1	3	5393.863	-0.000
4	0	4	3	0	3	5404.241	0.000
3	1	2	2	1	1	5481.484	0.000
3	2	1	2	2	0	5929.010	0.000
8	3	5	8	3	6	6387.251	-0.004
4	2	3	3	2	2	6490.119	0.001
7	2	5	7	2	6	6495.268	-0.002
7	3	5	7	1	6	6511.430	-0.003
6	1	5	6	1	6	6555.189	0.005
6	2	5	6	0	6	6557.958	0.006
5	1	5	4	1	4	6597.453	-0.000
5	0	5	4	0	4	6599.059	-0.000
4	1	3	3	1	2	6711.630	0.000
4	3	2	3	3	1	7161.483	0.000
7	1	6	7	1	7	7757.361	0.000
7	2	6	7	0	7	7757.774	-0.001
5	2	4	4	2	3	7772.480	-0.001
4	2	2	3	2	1	7781.419	0.002
6	1	6	5	1	5	7797.645	-0.000
6	0	6	5	0	5	7797.867	-0.000
4	3	1	3	3	0	7815.525	0.001
5	1	4	4	1	3	7837.714	-0.000

Table S2. Measured frequencies and residuals (MHz) of the rotational transitions of the $^{13}C_2$ isotopologue of 1,4-NQ.

J'	*K'*$_{-1}$	*K'*$_{+1}$	*J"*	*K"*$_{-1}$	*K"*$_{+1}$	ν_{obs}	ν_{obs}- ν_{calc}
2	1	2	1	1	1	2868.691	0.001
2	0	2	1	0	1	3042.175	-0.001
2	1	1	1	1	0	3838.530	0.001
3	1	3	2	1	2	4143.342	0.000
3	0	3	2	0	2	4201.757	0.000
3	2	2	2	2	1	5030.416	0.001
4	1	4	3	1	3	5358.059	0.000
4	0	4	3	0	3	5369.877	-0.000
3	1	2	2	1	1	5439.556	0.000
3	2	1	2	2	0	5859.072	0.000
4	2	3	3	2	2	6438.682	-0.000
5	1	5	4	1	4	6554.345	0.000
5	0	5	4	0	4	6556.267	-0.000
4	1	3	3	1	2	6674.359	-0.001
4	3	2	3	3	1	7086.536	-0.001
4	2	2	3	2	1	7708.558	-0.001
4	3	1	3	3	0	7709.915	-0.000
5	2	4	4	2	3	7718.256	0.002
6	1	6	5	1	5	7746.826	-0.000
6	0	6	5	0	5	7747.105	-0.000
5	1	4	4	1	3	7791.839	-0.000

TABLE S3. Measured frequencies and residuals (MHz) of the rotational transitions of the $^{13}C_1$ isotopologue of 1,4-NQ.

J'	*K'*$_{-1}$	*K'*$_{+1}$	*J"*	*K"*$_{-1}$	*K"*$_{+1}$	ν_{obs}	ν_{obs}- ν_{calc}
2	1	2	1	1	1	2883.396	0.001
2	0	2	1	0	1	3050.110	0.000
2	1	1	1	1	0	3868.919	0.000
3	1	3	2	1	2	4158.363	0.000
3	0	3	2	0	2	4210.696	0.001
3	2	2	2	2	1	5064.234	0.000
4	1	4	3	1	3	5374.344	0.000
4	0	4	3	0	3	5384.212	0.000
3	1	2	2	1	1	5463.360	0.000
3	2	1	2	2	0	5917.772	0.000
4	2	3	3	2	2	6469.372	-0.000
5	1	5	4	1	4	6573.363	-0.001
5	0	5	4	0	4	6574.863	-0.001
4	1	3	3	1	2	6684.872	-0.000
4	3	2	3	3	1	7144.756	-0.000
5	2	4	4	2	3	7745.188	-0.000
4	2	2	3	2	1	7760.123	0.002
6	1	6	5	1	5	7769.135	0.002
6	0	6	5	0	5	7769.337	-0.000
4	3	1	3	3	0	7805.277	-0.001
5	1	4	4	1	3	7807.399	-0.001

TABLE S4. Measured frequencies and residuals (MHz) of the rotational transitions of the $^{13}C_9$ isotopologue of 1,4-NQ.

J'	K'_{-1}	K'_{+1}	*J"*	K''_{-1}	K''_{+1}	ν_{obs}	$\nu_{obs}-\nu_{calc}$
2	1	2	1	1	1	2891.049	-0.001
2	0	2	1	0	1	3059.494	0.001
2	1	1	1	1	0	3877.423	-0.001
3	1	3	2	1	2	4170.439	0.001
3	0	3	2	0	2	4223.942	-0.000
3	2	2	2	2	1	5076.355	-0.000
4	1	4	3	1	3	5390.462	0.000
4	0	4	3	0	3	5400.670	0.001
3	1	2	2	1	1	5478.619	-0.000
3	2	1	2	2	0	5928.768	-0.000
4	2	3	3	2	2	6486.965	-0.000
5	1	5	4	1	4	6593.219	0.000
5	0	5	4	0	4	6594.789	0.000
4	1	3	3	1	2	6706.549	-0.001
4	3	2	3	3	1	7160.109	-0.000
5	2	4	4	2	3	7767.866	0.001
4	2	2	3	2	1	7778.871	0.001
6	1	6	5	1	5	7792.626	-0.001
6	0	6	5	0	5	7792.842	-0.001
4	3	1	3	3	0	7816.765	-0.000
5	1	4	4	1	3	7832.095	0.000

TABLE S5. Measured frequencies and residuals (MHz) of the rotational transitions of the $^{13}C_8$ isotopologue of 1,4-NQ.

J'	K'_{-1}	K'_{+1}	*J"*	K''_{-1}	K''_{+1}	ν_{obs}	$\nu_{obs}-\nu_{calc}$
2	1	2	1	1	1	2878.162	0.002
2	0	2	1	0	1	3046.457	-0.001
2	1	1	1	1	0	3859.304	0.001
3	1	3	2	1	2	4152.332	-0.001
3	0	3	2	0	2	4206.088	0.000
3	2	2	2	2	1	5053.096	-0.000
4	1	4	3	1	3	5367.300	-0.000
4	0	4	3	0	3	5377.613	-0.000
3	1	2	2	1	1	5454.542	0.000
3	2	1	2	2	0	5900.105	0.001
4	2	3	3	2	2	6458.236	-0.000
5	1	5	4	1	4	6564.959	0.002
5	0	5	4	0	4	6566.549	-0.002
4	1	3	3	1	2	6678.511	-0.001
4	3	2	3	3	1	7126.477	-0.000
5	2	4	4	2	3	7734.228	-0.000
4	2	2	3	2	1	7743.297	0.000
6	1	6	5	1	5	7759.236	0.001
6	0	6	5	0	5	7759.455	-0.001
4	3	1	3	3	0	7777.547	-0.001
5	1	4	4	1	3	7799.063	0.000

TABLE S6. Measured frequencies and residuals (MHz) of the rotational transitions of the $^{13}C_7$ isotopologue of 1,4-NQ.

J'	K'_{-1}	K'_{+1}	*J"*	K''_{-1}	K''_{+1}	ν_{obs}	$\nu_{obs}-\nu_{calc}$
2	1	2	1	1	1	2859.046	0.001
2	0	2	1	0	1	3034.489	-0.001
2	1	1	1	1	0	3821.962	-0.000
3	1	3	2	1	2	4131.501	-0.000
3	0	3	2	0	2	4191.971	0.000
3	2	2	2	2	1	5010.754	-0.000
4	1	4	3	1	3	5343.849	0.000
4	0	4	3	0	3	5356.374	-0.000
3	1	2	2	1	1	5422.388	-0.001
3	2	1	2	2	0	5829.538	0.000
4	2	3	3	2	2	6417.706	-0.001
5	1	5	4	1	4	6537.294	0.000
5	0	5	4	0	4	6539.377	-0.001
4	1	3	3	1	2	6659.910	-0.001
4	3	2	3	3	1	7055.245	0.001
4	3	1	3	3	0	7665.012	-0.000
4	2	2	3	2	1	7678.084	-0.000
5	2	4	4	2	3	7696.511	0.001
6	1	6	5	1	5	7726.739	0.000
6	0	6	5	0	5	7727.049	-0.001
5	1	4	4	1	3	7774.144	0.001

TABLE S7. Measured frequencies and residuals (MHz) of the rotational transitions of the ^{18}O isotopologue of 1,4-NQ.

J'	K'_{-1}	K'_{+1}	*J"*	K''_{-1}	K''_{+1}	ν_{obs}	$\nu_{obs}-\nu_{calc}$
2	1	2	1	1	1	2852.255	-0.003
2	0	2	1	0	1	3000.406	0.001
2	1	1	1	1	0	3848.963	-0.001
3	1	3	2	1	2	4100.236	-0.003
3	0	3	2	0	2	4139.913	-0.001
3	2	2	2	2	1	5025.916	0.000
4	1	4	3	1	3	5293.202	0.001
4	0	4	3	0	3	5299.587	0.001
3	1	2	2	1	1	5391.918	-0.001
3	2	1	2	2	0	5911.915	-0.003
4	2	3	3	2	2	6392.987	0.001
5	1	5	4	1	4	6472.577	-0.001
5	0	5	4	0	4	6473.411	-0.001
4	1	3	3	1	2	6562.945	-0.000
4	3	2	3	3	1	7112.534	-0.000
5	2	4	4	2	3	7634.142	0.001
6	1	6	5	1	5	7649.760	0.001
6	0	6	5	0	5	7649.858	0.002
5	1	4	4	1	3	7675.342	-0.001
4	2	2	3	2	1	7694.185	0.003
4	3	1	3	3	0	7836.198	0.001

Table S8. r_s atomic coordinates calculated from different pairs of rotational constants of 1,4-NQ, and their average values.

	A+B		A+C		B+C		Average	
	|a|/Å	|b|/Å	|a|/Å	|b|/Å	|a|/Å	|b|/Å	|a|/Å	|b|/Å
$^{13}C_{2/3}$	2.2580(7)	0.6685(3)	2.2576(7)	0.6686(2)	2.2581(7)	0.6668(3)	2.2579(7)	0.6680(3)
$^{13}C_{1/4}$	0.9944(5)	1.4533(10)	0.9945(5)	1.4534(10)	0.9945(5)	1.4534(10)	0.9945(5)	1.4534(10)
$^{13}C_{9/10}$	0.2537(60)	0.6930(2)	0.2527(60)	0.6930(2)	0.2537(60)	0.6926(2)	0.2534(60)	0.6929(2)
$^{13}C_{8/5}$	1.4911(10)	1.4002(11)	1.4907(10)	1.4003(11)	1.4912(10)	1.4000(11)	1.4910(10)	1.4002(10)
$^{13}C_{7/6}$	2.6960(6)	0.6976(2)	2.6960(6)	0.6978(2)	2.6960(6)	0.6978(2)	2.6960(6)	0.6977(2)
^{18}O	1.0341(5)	2.6759(6)	1.0332(5)	2.6761(6)	1.0347(5)	2.6755(6)	1.0340(5)	2.6758(6)

Errors include Costain's error.

Table S9. Equilibrium rotational constants $B_e^{calc.}$, and ground state constants $B_0^{calc.}$ from B3LYP/6-311++G(d,p) anharmonic frequency calculations, and semi-experimental equilibrium rotational constants B_e^{SE} of 1,4-NQ.

		$B_e^{calc.}$/MHz	$B_0^{calc.}$/MHz	B_e^{SE}/MHz
Parent	A	1326.154	1318.506	1335.940
	B	1092.375	1085.688	1099.493
	C	598.983	595.689	603.146
$^{13}C_2$	A	1324.690	1317.021	1334.498
	B	1080.458	1073.902	1087.419
	C	595.087	591.825	599.204
$^{13}C_1$	A	1318.876	1311.289	1328.656
	B	1089.958	1083.329	1097.049
	C	596.770	593.504	600.924
$^{13}C_9$	A	1324.440	1316.879	1334.185
	B	1092.183	1085.500	1099.336
	C	598.575	595.301	602.740
$^{13}C_8$	A	1319.591	1312.115	1329.160
	B	1087.005	1080.300	1094.174
	C	596.029	592.763	600.163
$^{13}C_7$	A	1324.601	1317.008	1334.331
	B	1075.401	1068.799	1082.457
	C	593.532	590.268	597.661
^{18}O	A	1279.843	1272.527	1289.295
	B	1086.333	1079.723	1093.439
	C	587.588	584.379	591.686

Table S10. r_e^{SE} atomic coordinates calculated from different pairs of rotational constants of 1,4-NQ, and their average values.

	A+B		A+C		B+C		Average	
	|a|/Å	|b|/Å	|a|/Å	|b|/Å	|a|/Å	|b|/Å	|a|/Å	|b|/Å
$^{13}C_{2/3}$	2.2568(7)[a]	0.6600(3)	2.2567(7)	0.6600(3)	2.2568(7)	0.6598(3)	2.2567(7)	0.6599(3)
$^{13}C_{1/4}$	1.0003(2)	1.4513(10)	1.0003(5)	1.4513(10)	1.0003(5)	1.4513(10)	1.0003(5)	1.4513(10)
$^{13}C_{9/10}$	0.2554(9)	0.7068(21)	0.2579(8)	0.7068(21)	0.2554(9)	0.7077(21)	0.2562(9)	0.7071(21)
$^{13}C_{8/5}$	1.4791(10)	1.4101(11)	1.4793(10)	1.4102(11)	1.4791(10)	1.4104(11)	1.4792(10)	1.4102(10)
$^{13}C_{7/6}$	2.6860(6)	0.7058(21)	2.6860(6)	0.7058(21)	2.6860(6)	0.7059(21)	2.6860(6)	0.7058(21)
^{18}O	1.0341(5)	2.6705(6)	1.0338(5)	2.6705(6)	1.0342(5)	2.6703(6)	1.0340(5)	2.6704(6)

[a] Errors include Costain's error.

Table S11. Least squares fit (last iteration) of the mass-weighted r_m structure of 1,4-NQ.

```
 ____________________________________________________________________________
|                                                                            |
|    STRFIT - General structure fitting program using CART definitions       |
|____________________________________________________________________________|
 version 25.VII.2019                                          Zbigniew KISIEL

______________________________________________________________________________

naphthoquinone
______________________________________________________________________________


NUMBER OF ATOMS = 18

  NO  NA  NB  NC       NO.NA      NO.NA.NB    NO.NA.NB.NC        MASS

   1   0   0   0    0.000000     0.000000      0.000000     12.0000000
   2   1   0   0    1.483885     0.000000      0.000000     12.0000000
   3   1   2   0    1.492400   117.247000      0.000000     12.0000000
   4   2   1   3    1.339937   122.232000     -0.011000     12.0000000
   5   3   1   2    1.406973   120.521000      0.009000     12.0000000
   6   4   2   1    1.483881   122.232000      0.007000     12.0000000
   7   1   2   3    1.220115   120.445000    180.003000     15.9949146
   8   3   1   5    1.396762   119.668000    179.997000     12.0000000
   9   8   3   5    1.391041   120.006000     -0.001000     12.0000000
  10   9   8   3    1.397095   120.183000      0.001000     12.0000000
  11   5   3  10    1.396769   119.813000      0.000000     12.0000000
  12   6   5   4    1.220104   122.308000    179.997000     15.9949146
  13   8   9   3    1.083186   121.346000    179.999000      1.0078250
  14   2   1   4    1.084734   115.483000    180.006000      1.0078250
  15   4   6   2    1.084738   115.483000    180.003000      1.0078250
  16  11   5  10    1.083183   118.649000    180.000000      1.0078250
  17  10  11   9    1.084032   119.866000    180.000000      1.0078250
  18   9  10   8    1.084034   119.950000    180.001000      1.0078250

---------------------------------------------
TOTAL NUMBER OF STRUCTURAL PARAMETERS:    11
---------------------------------------------

Parameters to be fitted:
          R( 2, 1) =     1.483885
                          , and at 1 more atom(s):   6
          R( 3, 1) =     1.492400
          R( 4, 2) =     1.339937
          R( 5, 3) =     1.406973
          R( 7, 1) =     1.220115
                          , and at 1 more atom(s):  12
          R( 8, 3) =     1.396762
                          , and at 1 more atom(s):  11

          R( 9, 8) =     1.391041
          R(10, 9) =     1.397095
              c_a  =     0.000000
              d_a  =     0.000000
              d_c  =     0.000000
 ------------------------------------------
```

TOTAL NUMBER OF SPECTROSCOPIC CONSTANTS: 21

Isotopic species		B_expt	Ib_expt	dI (g_bb)	dB	dB_el	B_corr	Ib_corr
	A	1328.29099	380.47311	0.00000	0.00000	0.00000	1328.29099	380.47311
	B	1092.80407	462.46077	0.00000	0.00000	0.00000	1092.80407	462.46077
	C	599.85210	842.50603	0.00000	0.00000	0.00000	599.85210	842.50603
2	A	1326.83400	380.89091	0.00000	0.00000	0.00000	1326.83400	380.89091
	B	1080.86109	467.57073	0.00000	0.00000	0.00000	1080.86109	467.57073
	C	595.94222	848.03357	0.00000	0.00000	0.00000	595.94222	848.03357
3	A	1321.06385	382.55457	0.00000	0.00000	0.00000	1321.06385	382.55457
	B	1090.41877	463.47241	0.00000	0.00000	0.00000	1090.41877	463.47241
	C	597.65875	845.59794	0.00000	0.00000	0.00000	597.65875	845.59794
4	A	1326.61981	380.95241	0.00000	0.00000	0.00000	1326.61981	380.95241
	B	1092.65186	462.52519	0.00000	0.00000	0.00000	1092.65186	462.52519
	C	599.46628	843.04826	0.00000	0.00000	0.00000	599.46628	843.04826
5	A	1321.67800	382.37680	0.00000	0.00000	0.00000	1321.67800	382.37680
	B	1087.46717	464.73036	0.00000	0.00000	0.00000	1087.46717	464.73036
	C	596.89758	846.67626	0.00000	0.00000	0.00000	596.89758	846.67626
6	A	1326.72340	380.92266	0.00000	0.00000	0.00000	1326.72340	380.92266
	B	1075.85715	469.74546	0.00000	0.00000	0.00000	1075.85715	469.74546
	C	594.39712	850.23799	0.00000	0.00000	0.00000	594.39712	850.23799
7	A	1281.97758	394.21829	0.00000	0.00000	0.00000	1281.97758	394.21829
	B	1086.82841	465.00350	0.00000	0.00000	0.00000	1086.82841	465.00350
	C	588.57652	858.64623	0.00000	0.00000	0.00000	588.57652	858.64623

```
    B_corr = B_expt + dB - dB_el, Ib_corr=505379.01/B_corr
or Ib_corr = Ib_expt + dI,        B_corr =505379.01/Ib_corr
```

DEFINITIONS OF SUBSTITUTED ISOTOPIC SPECIES

```
!
!  species 2  = 13C-1
!
ISOTOPIC SPECIES  2,  changes from parent species:
atom no.,parameter no.,value     4  4       13.0033548
!
!  species 3  = 13C-2
!
ISOTOPIC SPECIES  3,  changes from parent species:
atom no.,parameter no.,value     6  4       13.0033548
!
!  species 4  = 13C-3
!
ISOTOPIC SPECIES  4,  changes from parent species:
atom no.,parameter no.,value     5  4       13.0033548
!
!  species 5  = 13C-4
!
ISOTOPIC SPECIES  5,  changes from parent species:
atom no.,parameter no.,value    11  4       13.0033548
!
!  species 6  = 13C-5
!
ISOTOPIC SPECIES  6,  changes from parent species:
atom no.,parameter no.,value    10  4       13.0033548
!
!  species 7  = 18O
!
ISOTOPIC SPECIES  7,  changes from parent species:
atom no.,parameter no.,value    12  4       17.9991610
```

```
after:    7 iterations,  ALAMDA=  0.10E-09

 FINAL RESULTS OF LEAST SQUARES FIT:

          R( 2, 1) =     1.489995 +-  0.002910      and at atom  6
          R( 3, 1) =     1.481427 +-  0.003215
          R( 4, 2) =     1.304476 +-  0.007399
          R( 5, 3) =     1.381132 +-  0.008057
          R( 7, 1) =     1.214014 +-  0.003029      and at atom 12
          R( 8, 3) =     1.407615 +-  0.004082      and at atom 11
          R( 9, 8) =     1.387213 +-  0.002957
          R(10, 9) =     1.387419 +-  0.007820
              c_a  =     0.451802 +-  0.052294
              d_a  =    -1.419969 +-  0.568083
              d_c  =     2.520169 +-  0.481608

              Chi-squared =        0.0008357599
         Deviation of fit =        0.009142

Ni Axis     Iobs        Icalc      Io-c             Bobs       Bcalc      Bo-c

 1  a    380.47311   380.48155  -0.00844        1328.2910   1328.2615    0.0294
 1  b    462.46077   462.45222   0.00855        1092.8041   1092.8243   -0.0202
 1  c    842.50603   842.49909   0.00693         599.8521    599.8570   -0.0049
 2  a    380.89091   380.87583   0.01508        1326.8340   1326.8865   -0.0525
 2  b    467.57073   467.57190  -0.00117        1080.8611   1080.8584    0.0027
 2  c    848.03357   848.02627   0.00730         595.9422    595.9474   -0.0051
 3  a    382.55457   382.56028  -0.00571        1321.0639   1321.0441    0.0197
 3  b    463.47241   463.47857  -0.00617        1090.4188   1090.4043    0.0145
 3  c    845.59794   845.59797  -0.00003         597.6588    597.6587    0.0000
 4  a    380.95241   380.95324  -0.00083        1326.6198   1326.6169    0.0029
 4  b    462.52519   462.52856  -0.00337        1092.6519   1092.6439    0.0080
 4  c    843.04826   843.05971  -0.01144         599.4663    599.4581    0.0081
 5  a    382.37680   382.37463   0.00217        1321.6780   1321.6855   -0.0075
 5  b    464.73036   464.73539  -0.00503        1087.4672   1087.4554    0.0118
 5  c    846.67626   846.67100   0.00526         596.8976    596.9013   -0.0037
 6  a    380.92266   380.92625  -0.00359        1326.7234   1326.7109    0.0125
 6  b    469.74546   469.74067   0.00479        1075.8572   1075.8681   -0.0110
 6  c    850.23799   850.24471  -0.00673         594.3971    594.3924    0.0047
 7  a    394.21829   394.21698   0.00131        1281.9776   1281.9818   -0.0042
 7  b    465.00350   465.00113   0.00236        1086.8284   1086.8339   -0.0055
 7  c    858.64623   858.64751  -0.00128         588.5765    588.5756    0.0009

 Correlation coefficients:

                           1      2      3      4      5      6      7      8

 1:          R( 2, 1)   1.000
 2:          R( 3, 1)  -0.245  1.000
 3:          R( 4, 2)  -0.286  0.324  1.000
 4:          R( 5, 3)   0.404  0.079  0.687  1.000
 5:          R( 7, 1)   0.011 -0.357 -0.906 -0.769  1.000
 6:          R( 8, 3)  -0.340 -0.701 -0.200 -0.443  0.377  1.000
 7:          R( 9, 8)   0.254 -0.215  0.094  0.362 -0.156 -0.409  1.000
 8:          R(10, 9)   0.053 -0.260  0.605  0.589 -0.534  0.361 -0.294  1.000
 9:              c_a   -0.219  0.172 -0.058 -0.160 -0.097 -0.062 -0.025 -0.280
10:              d_a    0.001 -0.094 -0.639 -0.639  0.731  0.150 -0.115 -0.467
11:              d_c   -0.214  0.060 -0.802 -0.902  0.757  0.114 -0.159 -0.821

                           9     10     11

 9:              c_a    1.000
10:              d_a   -0.579  1.000
11:              d_c    0.311  0.594  1.000
```

Final principal coordinates of parent:

ATOM NO.	A	B	C	MASS
1	0.996034	-1.447128	0.000024	12.0000000
2	2.257984	-0.654937	-0.000113	12.0000000
3	-0.278640	-0.692255	-0.000024	12.0000000
4	2.260566	0.649536	-0.000056	12.0000000
5	-0.275906	0.688875	-0.000001	12.0000000
6	1.001761	1.446716	-0.000005	12.0000000
7	1.031490	-2.660624	0.000112	15.9949146
8	-1.501367	-1.389618	-0.000028	12.0000000
9	-2.699125	-0.689806	-0.000031	12.0000000
10	-2.696379	0.697610	-0.000009	12.0000000
11	-1.495839	1.391115	0.000006	12.0000000
12	1.036377	2.660237	0.000085	15.9949146
13	-1.481526	-2.472623	-0.000045	1.0078250
14	3.173871	-1.236141	-0.000159	1.0078250
15	3.178750	1.227112	-0.000095	1.0078250
16	-1.471692	2.474029	0.000024	1.0078250
17	-3.634035	1.241601	-0.000003	1.0078250
18	-3.639468	-1.229144	-0.000034	1.0078250

Principal coordinates and estimated uncertainties:

ATOM NO.	A	dA	B	dB	C	dC
1	0.99603	0.00162	-1.44713	0.00358	0.00002	0.00000
2	2.25798	0.00160	-0.65494	0.00379	-0.00011	0.00000
3	-0.27864	0.00224	-0.69225	0.00369	-0.00002	0.00000
4	2.26057	0.00147	0.64954	0.00423	-0.00006	0.00000
5	-0.27591	0.00316	0.68887	0.00459	0.00000	0.00000
6	1.00176	0.00210	1.44672	0.00365	-0.00001	0.00000
7	1.03149	0.00261	-2.66062	0.00140	0.00011	0.00000
8	-1.50137	0.00240	-1.38962	0.00380	-0.00003	0.00000
9	-2.69912	0.00156	-0.68981	0.00439	-0.00003	0.00000
10	-2.69638	0.00117	0.69761	0.00435	-0.00001	0.00000
11	-1.49584	0.00194	1.39112	0.00373	0.00001	0.00000
12	1.03638	0.00176	2.66024	0.00147	0.00008	0.00000
13	-1.48153	0.00329	-2.47262	0.00380	-0.00005	0.00000
14	3.17387	0.00196	-1.23614	0.00414	-0.00016	0.00000
15	3.17875	0.00176	1.22711	0.00472	-0.00009	0.00000
16	-1.47169	0.00269	2.47403	0.00373	0.00002	0.00000
17	-3.63403	0.00164	1.24160	0.00540	0.00000	0.00000
18	-3.63947	0.00198	-1.22914	0.00492	-0.00003	0.00000

NOTES: 1/ only the uncertainties for those coordinates which are completely defined by the fitted internals should be trusted
2/ the uncertainties are somewhat limited by the linear approximation coord=(d coord/d parameter)*parameter used for evaluation
3/ only the effect of the internals R, A, and D is propagated

---

Terms in I.fitted = I.rigid + eps

Ni	(I_a)rig	(I_b)rig	(I_c)rig	eps_a	eps_b^**	eps_c
1	374.73086	462.45222	837.18308	5.75069	0.00000	5.31602
2	375.12831	467.57037	842.69867	5.74753	0.00154	5.32760
3	376.79352	463.47685	840.27038	5.76676	0.00172	5.32760
4	375.20358	462.52853	837.73211	5.74966	0.00003	5.32760
5	376.61143	464.73197	841.34340	5.76321	0.00342	5.32760
6	375.17886	469.73826	844.91711	5.74740	0.00241	5.32760
7	388.34643	464.96855	853.31498	5.87055	0.03259	5.33253

** Only this value is the total EPSILON for the degenerate constant in linear and symmetric tops, the other value does not contain the rm() c and d contributions.

TABLE S12. Cartesian coordinates (in Angstroms) of the optimized geometry of naphthoquinone with different computational methods.

B3LYP/6-311++G(d,p)

1	C	0.000000	0.000000	0.000000
2	C	0.000000	0.000000	1.483885
3	C	1.326809	0.000000	-0.683254
4	C	1.133441	-0.000226	2.198548
5	C	2.516955	-0.000184	0.067161
6	C	2.472374	-0.000338	1.558891
7	O	-1.051880	0.000052	-0.618246
8	C	1.385827	0.000127	-2.078769
9	C	2.618747	0.000104	-2.722899
10	C	3.800538	-0.000070	-1.977751
11	C	3.750749	-0.000224	-0.587602
12	O	3.483655	-0.000605	2.241507
13	H	0.458030	0.000255	-2.637771
14	H	-0.979207	0.000097	1.950578
15	H	1.134431	-0.000290	3.283285
16	H	4.655081	-0.000373	0.008611
17	H	4.759317	-0.000089	-2.483583
18	H	2.661925	0.000211	-3.806072

MP2/6-311++G(d,p)

1	C	0.000000	0.000000	0.000000
2	C	0.000000	0.000000	1.483200
3	C	1.325515	0.000000	-0.684962
4	C	1.144856	0.001403	2.201479
5	C	2.519784	0.001172	0.064760
6	C	2.480690	0.001800	1.555910
7	O	-1.057767	-0.000427	-0.625512
8	C	1.379557	-0.000632	-2.087054
9	C	2.615600	-0.000354	-2.736230
10	C	3.805106	0.000689	-1.989638
11	C	3.758388	0.001402	-0.594311
12	O	3.504025	0.002757	2.236203
13	H	0.447629	-0.001377	-2.645164
14	H	-0.977983	-0.000417	1.958663
15	H	1.142647	0.001750	3.288876
16	H	4.666293	0.002191	0.002087
17	H	4.765005	0.000893	-2.498978
18	H	2.656701	-0.000904	-3.822117

M062X/6-311++G(d,p)

1	C	0.000000	0.000000	0.000000
2	C	0.000000	0.000000	1.487687
3	C	1.326465	0.000000	-0.686356
4	C	1.130012	-0.000137	2.197094
5	C	2.511493	-0.000099	0.057586
6	C	2.469821	-0.000232	1.550515

7	O	-1.041880	-0.000041	-0.614793
8	C	1.377166	0.000076	-2.077684
9	C	2.607222	0.000085	-2.721995
10	C	3.788304	-0.000001	-1.980540
11	C	3.742542	-0.000102	-0.592719
12	O	3.476313	-0.000431	2.221642
13	H	0.446297	0.000136	-2.632020
14	H	-0.980003	0.000056	1.952177
15	H	1.137590	-0.000164	3.281584
16	H	4.646390	-0.000181	0.004655
17	H	4.745243	0.000005	-2.488550
18	H	2.648943	0.000154	-3.804624
